# Concomitant Administration of Red Ginseng Extract with Lactic Acid Bacteria Increases the Plasma Concentration of Deglycosylated Ginsenosides in Healthy Human Subjects

**DOI:** 10.3390/biom12121896

**Published:** 2022-12-17

**Authors:** Ji-Hyeon Jeon, Jin-Hyang Park, So Yeon Jeon, Minyeong Pang, Min-Koo Choi, Im-Sook Song

**Affiliations:** 1 BK21 FOUR Community-Based Intelligent Novel Drug Discovery Education Unit, Vessel-Organ Interaction Research Center (VOICE), Research Institute of Pharmaceutical Sciences, College of Pharmacy, Kyungpook National University, Daegu 41566, Republic of Korea; 2 College of Pharmacy, Dankook University, Cheon-an 31116, Republic of Korea

**Keywords:** deglycosylation metabolism, ginsenosides, lactic acid bacteria (LAB), pharmacokinetics, red ginseng extract (RGE)

## Abstract

With the increased frequency of red ginseng extract (RGE) and lactic acid bacteria (LAB) co-administration, we aimed to investigate the interactions between RGE and LAB with regard to in vitro and in vivo deglycosylation metabolism and the pharmacokinetics of ginsenosides. As a proof-of-concept study, five healthy humans were administered RGE (104.1 mg of total ginsenosides/day) with or without co-administration of LAB (2 g, 1 billion CFU/day) for 2 weeks, and the plasma concentrations of ginsenosides in human plasma were monitored. The plasma exposure to compound K (CK), ginsenoside Rh2 (GRh2), protopanaxadiol (PPD), and protopanaxatriol (PPT) in the concomitant administration RGE and LAB groups increased by 2.7-, 2.1-, 1.6-, and 3.5-fold, respectively, compared to those in the RGE administration group, without a significant change in T_max_. The plasma concentrations of GRb1, GRb2, and GRc remained unchanged, whereas the AUC values of GRd and GRg3 significantly decreased in the concomitant administration RGE and LAB groups. To understand the underlying mechanism, the in vitro metabolic activity of ginsenosides was measured during the fermentation of RGE or individual ginsenosides in the presence of LAB for 1 week. Consistent with the in vivo results, co-incubation with RGE and LAB significantly increased the formation rate of GRh2, CK, PPD, and PPT. These results may be attributed to the facilitated deglycosylation of GRd and GRg3 and the increased production of GRh2, CK, PPD, and PPT by the co-administration of LAB and RGE. In conclusion, LAB supplementation increased the plasma concentrations of deglycosylated ginsenosides, such as GRh2, CK, PPD, and PPT, through facilitated deglycosylation metabolism of ginsenosides in the intestine.

## 1. Introduction

Among herbal medicines, red ginseng extract (RGE) is widely known. Efficacy studies have indicated that ginsenoside components are responsible for RGE activity [1,2,3]. Depending on the position and number of sugar chains and hydroxyl groups on the dammarane structure, ginsenosides are divided into two types: 20(s)-protopanaxadiol (PPD)-type and 20(s)-protopanaxatriol (PPT)-type. PPD-type ginsenosides may be further subdivided into ginsenoside Rb1 (GRb1), GRb2, GRc, GRd, GF2, GRg3, compound K (CK), GRh2, and PPD, according to their glycosylation statuses. Similarly, PPT-type ginsenosides may be subdivided into GRe, GRg1, GRf, GF1, GRh, and PPT, based on their glycosylation statuses. Ginseng products contain various ginsenosides; however, the ginsenoside content varies depending on the ginseng production process and plant origin [4]. RGE shows a higher proportion of PPD-type ginsenosides (2.2 ~ 5.4-fold) compared with that of the PPT-type ginsenosides and the highly glycosylated ginsenosides such as GRb1, GRb2, GRc, GRg1, and GRe constitute the major ginsenosides [5]. PPD-type ginsenosides were mainly detected in human plasma after oral administration of RGE [6,7].

GRb1, GRg3, GRg5, and CK are the predominant ginsenosides in black ginseng extract [8]. Therefore, the plasma GRg3 concentrations in humans treated with black ginseng extract were much higher than those in humans treated with RGE [9]. We previously reported that the PPD-type ginsenosides GRb1, GRb2, GRc, and GRd were detectable in rat plasma, and the area under the concentration curve (AUC) values of GRb1, GRb2, GRc, and GRd were correlated with their content in ginseng products [10]. Kim et al. [11] reported that the oral intake of GRg3, GRd, CK, and GF2 correlates with their plasma concentrations in humans. These results suggest that the absorption of ginsenosides is dependent on the content of the ginseng product [10].

Recently, there have been reports on contributing factors that affect the pharmacokinetics of ginsenosides, except for the ginsenoside content in ginseng products. Jeon et al. [12] reported that supplementation of lactic acid bacteria (LAB) with RGE facilitated the deglycosylation of ginsenosides (i.e., the metabolic conversion of PPD from GRh2 or CK, the formation of PPT from GRh1 or GF1, and the formation of CK from GF2). These LAB-mediated ginsenoside metabolisms might increase the content of deglycosylated ginsenosides and, consequently, result in markedly higher AUC values of these ginsenosides in LAB-supplemented mice compared with that found in the gut microbiota-mediated intrinsic deglycosylation activity [13,14].

In addition, the lipophilicity of ginsenosides varies depending on the number of sugar chains (i.e., glycosylation states) and has different pharmacokinetic and pharmacological profiles [15]. Mono-glycosylated or aglycone ginsenosides are widely known to be readily absorbed into the bloodstream and act as active compounds [6,16,17]. For example, the oral bioavailability (BA) of highly glycosylated ginsenosides such as GRb1, GRb2, and GRd was less than 5% in rats, while the oral BA of PPD, a deglycosylated ginsenoside, was 36.8–48.1% [15]. In contrast, the respective oral BA of PPT and CK (de- and mono-glycosylated ginsenosides, respectively) were 3.7% and 4.3% [18,19]. These results suggested that an unknown absorption mechanism might be involved in the ginsenoside’s BA, in addition to its increased lipophilicity caused by deglycosylation. Yang et al. [20,21] found that CK AUC was 23.5-fold greater in P-glycoprotein (P-gp)-null mice following an oral dose of CK (10 mg/kg) than compared to that in wild-type mice, suggesting that the efflux of CK via P-gp may limit the oral absorption of CK. Except for CK, limited information regarding the absorption mechanism or involvement of P-gp is available in the literature.

Therefore, as a proof-of-concept study in humans, we aimed to investigate the effects of LAB on the deglycosylation metabolism of ginsenosides and their pharmacokinetic changes after the co-administration of RGE and LAB for 2 weeks in healthy male volunteers and to analyze the major factors involved in the intestinal absorption of ginsenosides. To expand our understanding of the absorption mechanisms of ginsenosides, we evaluated the intestinal permeability of ginsenosides and the involvement of P-gp, which may limit the absorption of these ginsenosides in CaCo-2 cells as well as P-gp-overexpressing cells (i.e., LLC-PK1-P-gp cells) [22].

## 2. Materials and Methods

### 2.1. Materials

Commercially available red ginseng extract (RGE; Hongsamjung All Day ^®^, Lot no. 61920692) was purchased from the Punggi Ginseng Cooperative Association (Youngjoo, Kyungpook, Republic of Korea). RGE was produced in facilities according to the current guidelines of the Korean Good Manufacturing Practice. Briefly, dried ginseng radix of *Panax ginseng C.A. Meyer* cultivated for 6 years at the Punggi ginseng farm (Youngjoo, Kyungpook, Republic of Korea) was extracted using 8 volumes of water at 85 °C for 8 h with 4 cycles of repeated extraction. The extracts were concentrated under reduced pressure at 65 °C to make 70–73 brix of concentrated RGE, followed by sterilization using an autoclave at 121 °C for 15 min. The ginsenoside content was analyzed using a liquid chromatography-tandem mass spectrometry (LC-MS/MS) system. PPD-type and PPT-type ginsenosides were purchased from the Ambo Institute (Daejeon, Republic of Korea).

A commercially available lactic acid bacterial formulation (LAB; Bifidus Premium ^®^, Lot No. AP001, total 1 billion CFU/2 g), which is a dried mixture of viable bacteria from *Bifidobacterium bifidum* BGN4 (50%), *Bifidobacterium longum* BORI (30%), *Lactobacillus acidophilus* AD031 (10%), and *Lactobacillus faecium* BH06 (10%), was purchased from Bifido Co. Ltd. (Hongcheon-si, Kangwon-do, Republic of Korea). The relative composition of bacterial strains is expressed in parentheses above.

Difco Lactobacilli MRS broth was procured from BD BioSciences (Sparks, MD, USA). CaCo-2 cells were purchased from the American Type Culture Collection (Rockville, MD, USA). The LLC-PK1-P-gp cells and collagen-coated permeable support (1.0 μm pore size) in 24-well plates were purchased from Corning Life Sciences (Tewksbury, MA, USA). All other solvents and chemicals used were of analytical or reagent grade, as appropriate.

### 2.2. In Vitro RGE Fermentation Study with LAB

To identify the effects of LAB on ginsenoside metabolism, an in vitro fermentation study was performed according to the method of Jeon et al. [12], with slight modifications. Briefly, RGE (10 mL, 1 pouch) and 2 g LAB (containing 1 billion CFU) were suspended in 10 mL autoclaved MRS broth (550 mg broth powder dissolved in 10 mL water). For the 15 ginsenosides, individual ginsenosides (10 μM each) and 2 g of LAB were suspended in 20 mL of autoclaved MRS broth (550 mg broth powder dissolved in 20 mL water). The mixture was incubated for 7 days in a shaking incubator at 37 °C and 300 rpm, and a 0.1 mL aliquot of the incubation mixture was collected on days 0, 3, 5, and 7. The incubation medium was diluted 100-fold with distilled water and stored at −80 °C to analyze ginsenosides using the LC-MS/MS system.

### 2.3. Pharmacokinetic Study

This proof-of-concept pharmacokinetic study protocol was approved by the Institutional Review Board of Kyungpook National University Hospital (KNUH, Daegu, Republic of Korea) (approval number: KNUH 2018–04–028–002; date of approval: 12 July 2018) and registered at the Clinical Research Information Service (https://cris.nih.go.kr; Registry No. KCT0003035, Accessed on 22 August 2022). All participants provided written informed consent for inclusion in the study.

A single-sequence study consisting of two periods was conducted on five healthy male subjects (body weight > 50 kg and age > 19 years) for 29 days. During the first period of the study (RGE treatment group), three pouches of RGE (30 mL, 104.1 mg of total ginsenosides) were administered to 5 subjects once daily for 14 days. On the 14th day, the subjects were admitted to the Clinical Trial Center of KNUH at 7 AM and underwent the last RGE administration at approximately 8–9 AM. Blood samples (approximately 3 mL) were collected at 0, 4, 6, 8, 10, 12, 13, 14, and 24 h after the last dose. A saline-locked angiocatheter was placed on the forearm of each subject’s hand. After discarding 1 mL of blood derived from the catheter, 3 mL of blood was drawn into a vacutainer tube containing heparin and centrifuged at 3000× *g* for 10 min. The supernatant plasma samples were stored at −80 °C until ginsenoside analysis.

In the second half of the study (RGE + LAB treatment group), 3 pouches of RGE (30 mL, 104.1 mg of total ginsenosides) and 1 pouch of LAB (1 billion CFU/2 g) were co-administered once daily for 14 days. On the 28 h day, the subjects were admitted to the Clinical Trial Center of KNUH at 7 AM and received the last red ginseng dose at approximately 8–9 AM. Blood samples (approximately 3 mL) were withdrawn at 0, 4, 6, 8, 10, 12, 13, 14, and 24 h intervals after the last dose and centrifuged at 3000× *g* for 10 min; the supernatant plasma samples were stored at −80 °C. During the clinical study, no subjects showed any clinically significant abnormalities, which were monitored by a detailed physical examination, a clinical lab test, and a 12-lead electrocardiograph.

### 2.4. The Transport of Ginsenosides in Caco-2 and LLC-PK1-P-gp Cells

CaCo-2 cells (passage no 41–43) were seeded onto collagen-coated permeable support (1.0 μm pore size) in a 24-well plate at a density of 2.5 × 10^4^ cells/insert and cultured for 21 days in Dulbecco’s modified eagle’s medium supplemented with 10% fetal bovine serum, 1% non-essential amino acids, and 1% penicillin-streptomycin at 37 °C in a humidified atmosphere (5% CO_2_/95% air). After confirming that the transepithelial electrical resistance (TEER) of the CaCo-2 cell monolayer was over 400 Ω·cm^2^, Apical to basal (A to B) transport and B to A transport of ginsenosides using 50 µM, GRb1 was investigated as previously described [12,22,23]. The same permeability study was performed using 50 µM GRb2, GRc, GRd, GRg3, GRh2, GF2, CK, PPD, GRe, GRf, GRg1, GRh1, GF1, or PPT.

LLC-PK1-P-gp cells (passage no. 25–28) were seeded onto collagen-coated permeable support (1.0 μm pore size) in 24-well plates at a density of 2.5 × 10^4^ cells/insert and grown for 5 days with TEER values of over 500 Ω·cm^2^ [24]. The A to B transport and B to A transport of GRg3, GRh2, CK, PPD, GRg1, GRh1, and PPT (20 μM each) were measured as previously described [12,22,23]. The concentration dependency in the B to A transport of GRh2, CK, PPD, and PPT was measured in the range of 6.25–200 μM for GRh2, CK, PPD, and PPT.

### 2.5. LC-MS/MS Analysis of Ginsenosides

Ginsenoside concentrations were determined with an Agilent 6470 triple quadrupole LC-MS/MS system (Agilent, Wilmington, DE, USA) using the previously published method by Jin et al. [7,25]: The concentrations of GRb1, GRb2, GRc, GRd, GRg3, GF2, GRe, GRf, GRg1, and GF1 were analyzed using the protein precipitation method, and the concentrations of GRh2, CK, PPD, GRh1, and PPT were analyzed using the liquid−liquid extraction method.

Briefly, to analyze GRb1, GRb2, GRc, GRd, GRe, GRf, GRg1, GRg3, GF1, and GF2, 400 μL of an internal standard (IS; 0.05 ng/mL berberine in methanol) was added to 100 μL of plasma. The mixture was vortexed for 15 min and centrifuged at 16,000× *g* for 5 min. After centrifugation, 400 μL of the supernatant was evaporated using a speed-dry vacuum concentrator (Christ, Osterode am Harz, Germany). The residue was reconstituted using 200 μL of 70% methanol supplemented with 0.1% formic acid, and a 10 μL aliquot was injected into the LC-MS/MS system. Chromatographic separation was performed using a Polar RP column (150 × 2.0 mm, 4.0 μm particle size; Phenomenex, Torrance, CA, USA) using a mobile phase consisting of 0.1% formic acid in water (phase A) and 0.1% formic acid in methanol (phase B). The following elution gradient was used: 69% of phase B for 0–2.0 min, 69–85% of phase B for 2.0–4.0 min, and 85–69% of phase B for 6.0–6.5 min. The flow rate was 0.27 mL/min.

To analyze GRh1, GRh2, CK, PPD, and PPT, 30 μL of IS (20 ng/mL ^13^C-caffeine in water) and 400 μL of methyl-tert-butyl ether were added to 100 μL of plasma samples. The mixture was vortexed for 10 min and centrifuged at 16,000× *g* for 5 min. After centrifugation, the samples were frozen at −80 °C for 4 h. The upper layer was transferred to a clean tube and evaporated. The residue was reconstituted using 200 μL of 80% methanol supplemented with 0.1% formic acid, and a 10 μL aliquot was injected into the LC-MS/MS system. Chromatographic separation was performed using a Luna C18 column (150 × 2.0 mm, 3.0 μm particle size; Phenomenex, Torrance, CA, USA) and using a mobile phase consisting of water containing 0.1% formic acid: methanol containing 0.1% formic acid = 8:92 (*v*/*v*) at a flow rate of 0.15 mL/min.

The quantification of ginsenosides was carried out using multiple reaction monitoring in positive ionization mode, as previously described [7,12,26]. The standard calibration curves for each ginsenoside were linear in human plasma spiked with a concentration range of 0.5–200 ng/mL. The intra- and inter-day precision and accuracy for 15 ginsenosides were below 15 %.

### 2.6. Data Analysis

To measure the metabolic activity of ginsenosides, the formation rate of ginsenosides (pmol/day) was calculated by dividing the slope of the regression line by the amount of deglycosylated ginsenosides versus the incubation time plot by the initial concentration of individual ginsenosides [12].

For the transport study of ginsenosides in CaCo-2 cells and LLC-PK1-P-gp cells, the ginsenoside transport rate in the A to B or B to A directions was calculated from the slope of the regression line from the mean transported amounts versus time plot. The apparent permeability (P_app_) was calculated using Equation (1) [27,28].

(1) $$P_{\mathrm{app}}\;(\times 10^{-6}\;\mathrm{cm}/s)=\frac{transport\;rate\;\left(\mathrm{nmol}/\min\right)}{concentration\left(\mu M\right)\times area\left(cm^{2}\right)\times 60s}$$

The efflux ratio was calculated by dividing the P_app_ of ginsenosides from the B to A direction by that from the A to B direction.

To measure the kinetic parameters of the concentration dependence on the P-gp-mediated ginsenoside efflux, the ginsenoside transport rate in the B to A direction (ν) versus the ginsenoside concentration (S) plot was fitted in the Michaelis–Menten Equation (2) [26] using Sigma Plot (version 12.0, Systat Software Inc. Chicago, IL, USA).

(2) $$\nu =\frac{V_{max}\times S}{K_{m}+S}$$

In Equation (2), V_max_ indicates the maximum velocity of the B to A transport rate of ginsenosides, and K_m_ represents the ginsenoside concentration that showed half-maximal velocity [27].

Finally, pharmacokinetic parameters were calculated using non-compartmental methods (WinNonlin version 5.1; Pharsight Co., Certara, NJ, USA). Statistical analysis was performed by Student’s t-test to compare the two groups using IBM SPSS Statistics for Windows (version 25.0; IBM Corp., Armonk, NY, USA). Differences were considered significant at *p* < 0.05.

## 3. Results

### 3.1. Effect of LAB Supplementation on the In Vitro Ginsenoside Metabolism and In Vivo Pharmacokinetics of Ginsenosides in Human Subjects

**Figure 1:**
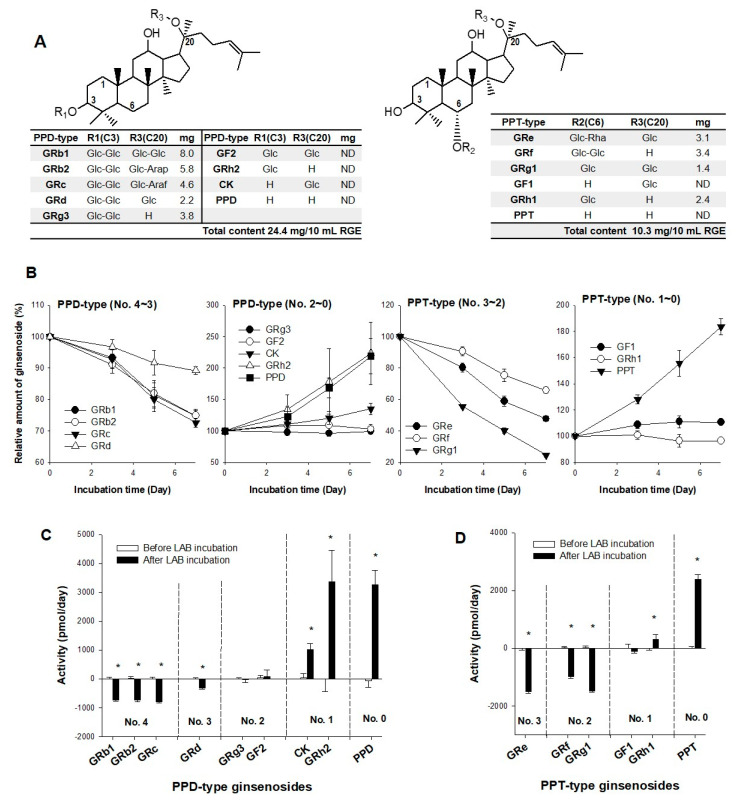
(**A**) Chemical structures of the PPD-type and PPT-type ginsenosides. Glc: glucose; Arap: arabinopyranose; Araf: arabinofuranose; Rha: rhamnose; Xyl: xylose. (**B**) Alterations of 15 ginsenosides following the incubation of RGE with LAB for 7 days are expressed as the relative percentage of ginsenoside. Metabolic activity of (**C**) the PPD-type ginsenosides and (**D**) the PPT-type ginsenosides following incubation of RGE (100 μg) with LAB (1 billion CFU), expressed as the alteration of the ginsenoside amount per day depending on the sugar number (No.) of the ginsenosides. Data points are represented as mean ± standard deviation (*n* = 4). * *p* < 0.05 compared with after LAB incubation group.

Among the 15 ginsenosides tested, RGE contained nine ginsenosides (Figure 1A). The highest abundance of ginsenosides was the tetra-glycosylated PPD-type (i.e., GRb1, GRb2, and GRc; 53% of total ginsenosides), and the ratio of PPD-type to PPT-type ginsenosides was 2.4 (Figure 1A), consistent with previous reports [4,5,26].

Following in vitro fermentation of RGE with LAB for 7 days, alterations in ginsenoside content were observed with increasing incubation time, depending on the number of sugar moieties or glycosylation status (Figure 1B). That is, the highly glycosylated ginsenosides, GRb1, GRb2, GRc, GRd (PPD-type ginsenosides with sugar No. 4–3), GRe, GRf, and GRg1 (PPT-type ginsenosides with sugar No. 3–2), exhibited a marked decrease following fermentation with LAB for 7 days (Figure 1B), and consequently showed negative metabolic activity (Figure 1C,D). The relative abundances of GRg3 and GF2 (PPD-type ginsenosides with sugar No. 2) and GF1 (PPT-type ginsenosides with sugar No. 1) remained unchanged, resulting in no significant metabolic activity (Figure 1C,D). In contrast, RGE incubation with LAB for 7 days significantly increased the relative abundance and formation rate of mono- or de-glycosylated ginsenosides (i.e., GRh2, CK, GRh1, PPD, and PPT; ginsenosides with sugar No. 1 or 0) (Figure 1B–D).

**Figure 2:**
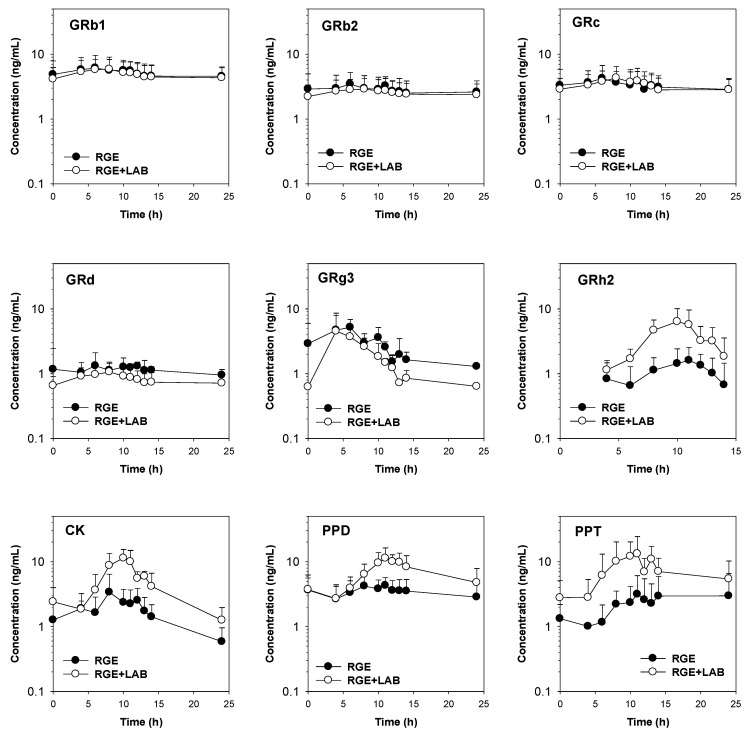
Plasma concentration-time profile of ginsenoside Rb1 (GRb1), GRb2, GRc, GRd, GRg3, GRh2, CK, PPD, and PPT in humans following the repeated oral administration of RGE for 14 days with or without repeated co-administration of LAB for 14 days (●: RGE; ○: RGE + LAB). The data points are represented as mean ± standard deviation (*n* = 5).

To determine the effect of LAB supplementation on the pharmacokinetics of ginsenosides, a proof-of-concept clinical study was performed to compare the pharmacokinetics of ginsenosides after repeated administration of RGE for 14 days, with or without LAB supplementation (Figure 2). The demographics of the five healthy male subjects enrolled in this study are summarized as follows: mean age, 24.6 years (range, 20–31 years); mean height, 172.9 cm (range, 165.3–181.3 cm); mean weight, 67.7 kg (range, 56.8–78.2 kg).

One pouch of the RGE used in this study contained 13.2 mg of marker ginsenosides (GRb1 + GRg1 + GRg3) and 34.7 mg of total ginsenosides. Therefore, the dose of RGE in this study was calculated as 39.6 mg of marker ginsenosides and 104.1 mg of total ginsenosides per day in accordance with the Criteria and Standards of Health Functional Food (MFDS Notice No. 2017-113). This ensured that the maximum daily intake of red ginseng should not exceed 80 mg of marker ginsenosides (GRb1 + GRg1 + GRg3). In addition, repeated oral doses of total ginsenosides ranging from 50 to 100 mg per day for more than 1 month were well tolerated with no adverse reports [4,16,29,30,31,32]. Clinical studies with high doses of RGE (3 g for 12 weeks, 4.5 g for 2 weeks, 6 g for 8 weeks, and 60 g for 11 days) reported no adverse effect, although the total ginsenoside content in these RGE doses was not identified [33]. Repeated administration of high-dose RGE: our study participants who took 104.1 mg of total ginsenosides for 29 days completed the pharmacokinetic study and safety assessment, and there were no severe or unexpected adverse events reported.

In human plasma samples, eight PPD-type ginsenosides (GRb1, GRb2, GRc, GRd, GRg3, GRh2, CK, and PPD) and only PPT were detected following repeated RGE administration (Figure 2). The pharmacokinetic parameters calculated from the plasma ginsenoside-time profiles are listed in Table 1.

The plasma concentrations and pharmacokinetic parameters of GRb1, GRb2, and GRc in subjects who ingested RGE and LAB were not different from those in the RGE group. The AUC values of GRd and GRg3 were significantly decreased in the RGE + LAB group but did not reach statistical significance for T_max_ or MRT compared with the RGE group (Table 1). The plasma concentrations of GRh2, CK, PPD, and PPT were enhanced by LAB supplementation. These results are consistent with the increased formation of GRh2, CK, PPD, and PPT by LAB incubation, suggesting a role of LAB in the deglycosylation metabolism of ginsenosides in humans. The AUC and C_max_ values of GRh2, CK, PPD, and PPT were significantly increased by RGE and LAB co-administration without significant alterations in T_max_ and MRT (Table 1). This may be attributed to the increased metabolic activity of CK, GRh2, PPD, and PPT due to LAB fermentation, as evidenced by the in vitro fermentation study.

**Figure 3:**
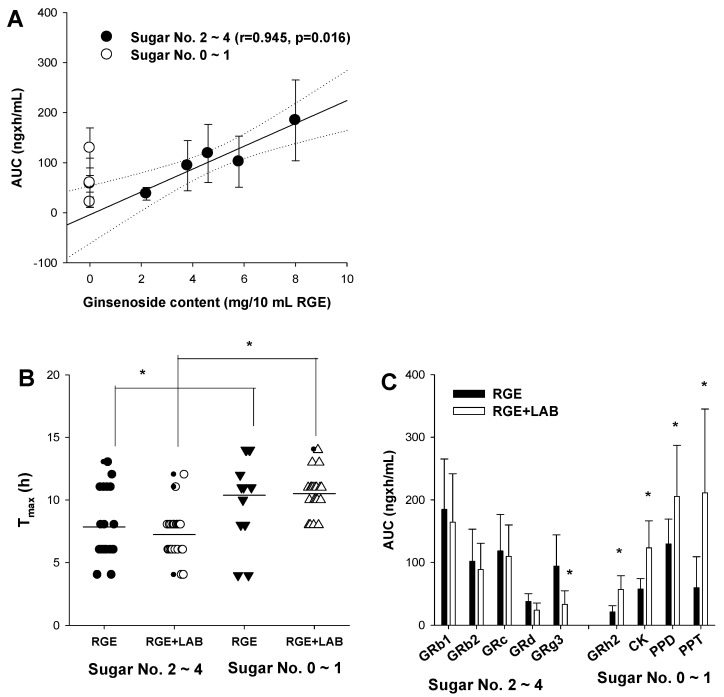
(**A**) Correlation between ginsenoside content and AUC values of ginsenosides after oral administration of RGE. The data are taken from Table 1 and Figure 1A. Lines were generated from the linear regression analysis and 90% confidence intervals. (**B**) T_max_ of ginsenosides was drawn depending on the sugar No. of the ginsenosides (● and ▼: RGE; ○ and ∆: RGE + LAB). (**C**) AUC of ginsenosides was drawn depending on the sugar No. of the ginsenosides; * *p* < 0.05 was considered statistically significant between the two groups; the data are taken from Table 1. Data points are represented as mean ± standard deviation (*n* = 5).

Correlation analysis of the AUC values of PPD-type ginsenosides and their content in RGE revealed that highly glycosylated ginsenosides with sugar numbers of 2–4 exhibited a good correlation (r = 0.945) and a *p*-value of 0.0162 (Figure 3A). This is consistent with the pharmacokinetic results in humans, rats, and mice [6,10,26]. In contrast, the AUC values of ginsenosides with sugar Nos. 0–1 did not correlate with their content because these ginsenosides were not abundant in RGE (Figure 3A). The presence of GRh2, CK, PPD, and PPT in human plasma may be attributed to intestinal metabolism [7,34,35,36]. We hypothesized that supplementation with LAB promotes ginsenoside deglycosylation metabolism in the intestine and consequently increases the plasma concentration of deglycosylated ginsenosides.

When compared with the T_max_ values of the ginsenosides relative to their glycosylation states, the T_max_ values of highly glycosylated ginsenosides with sugar Nos. 2–4 were significantly lower than those of deglycosylated ginsenosides with sugar Nos. 0–1, regardless of the LAB dosing regimen (Figure 3B). This suggests delayed absorption of GRh2, CK, PPD, and PPT after deglycosylation from highly glycosylated ginsenosides in the intestine. However, LAB supplementation to GRh2, CK, PPD, and PPT did not facilitate the absorption rate (T_max_) of deglycosylation (RGE group (▼) vs. RGE + LAB group (∆); *p* > 0.05). When compared with the AUC values of ginsenosides in relation to their glycosylation states, the AUC values of highly glycosylated ginsenosides with a sugar Nos. 2–4 were not altered or significantly decreased; however, the AUC values for GRh2, CK, PPD, and PPT (sugar Nos. 0–1) were 2.7-, 2.1-, 1.6-, and 3.5-fold, respectively, after LAB supplementation (Figure 3C). These results suggested that co-administration of RGE and LAB increases the production of GRh2, CK, PPD, and PPT with a limited effect on the absorption rate of ginsenoside deglycosylation.

### 3.2. LAB-Mediated Ginsenosides Metabolic Pathway

**Figure 4:**
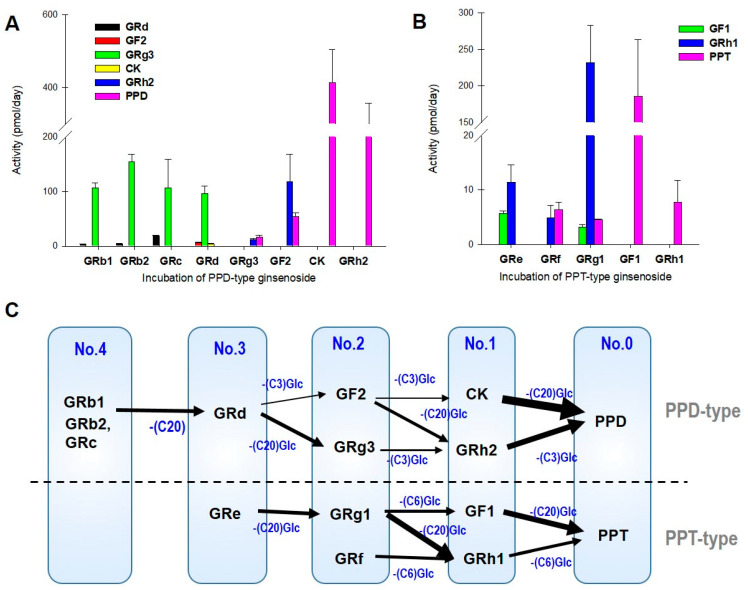
Metabolic activity of deglycosylated ginsenosides following incubation of each ginsenoside (10 μM each) with LAB (1 billion CFU) for 7 days. (**A**) PPD-type ginsenosides: GRb1, GRb2, GRc, GRd, GF2, GRg3, CK, and GRh2 and (**B**) PPT-type ginsenosides: GRf, GRe, GRg1, GF1, and GRh1 were incubated in the presence of LAB for 7 days. The data are expressed as mean ± standard deviation (*n* = 4). (**C**) PPD- and PPT-type ginsenosides were grouped by sugar number and deglycosylation pathway. Arrows indicate the relative deglycosylation activity at the −C3, −C6, or −C20 positions of ginsenosides by incubating with LAB from the results in (**A**,**B**). Glc; glucose residue.

We examined the LAB-mediated ginsenoside metabolic pathway by incubating individual ginsenosides (10 μM each) with LAB (Figure 4A,B). Incubation of GRb1, GRb2, GRc, and GRd with LAB resulted in GRg3 production having a higher formation rate than GF2. GRh2 and PPD were produced from GF2 at a higher formation rate than GRg3 (Figure 4A). The results suggested a higher deglycosylation rate at the C20 site than at the C3 site (Figure 4C). Moreover, deglycosylation of GF2 to CK was negligible after incubation for one week. The results also suggested greater deglycosylation at the C20 site of the damarane structure than at the C3 site mediated by LAB. The formation of PPD resulting from incubation with CK was the highest, and the formation of PPD from GRh2 was also higher than the other deglycosylation at the C3 site (Figure 4A). These results suggested that deglycosylation mediated by LAB is preferable at the C20 site of the ginsenoside structure compared with the C3 site, and deglycosylation from mono-glycosylated ginsenosides (CK and GRh2) is much greater than deglycosylation from a highly glycosylated state. This could explain the greater formation rate of PPD and GRh2 in RGE incubated with LAB (Figure 1), which is consistent with our previous results in mice and rats [26].

Similarly, incubation of GRf, GRe, and GRg1 (tri- or di-glycosylated PPT-type ginsenosides) with LAB produced a higher amount of GRh1 than GF1. Of these, the metabolic activity of GRh1 from GRg1 was the highest (Figure 4B), suggesting preferable deglycosylation mediated by LAB at the C20 site of the damarane structure compared to that at the C6 site (Figure 4C). The greater formation rate of PPT from GF1 compared with that of PPT from GRh1 (Figure 4B) also suggested preferable deglycosylation at the C20 site of the dammarane structure compared to the C6 site. Taken together, these results suggested that LAB fermentation induces stepwise and sequential deglycosylation of the dammarane structure by preferring the C20 site compared to the C3 or C6 site. In addition, the results suggested that the facilitated breakdown of ginsenosides from a large sugar number to deglycosylated ginsenosides with a smaller sugar number may change the ginsenoside content in the gut.

### 3.3. Permeability of Ginsenosides in CaCo-2 and LLC-PK1-P-gp Cells

**Figure 5:**
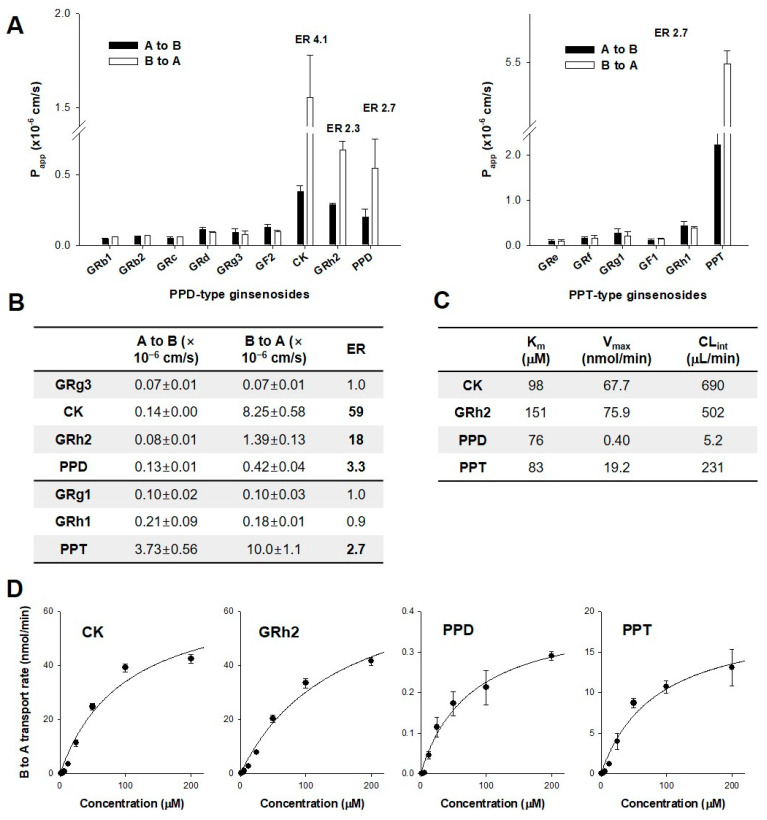
(**A**) The permeability (P_app_) of PPD-type ginsenosides and PPT-type ginsenosides from apical to basal (A to B) and B to A direction was measured in CaCo-2 cell monolayers. The efflux ratio (ER) was calculated by dividing B to A P_app_ by A to B P_app_. (**B**) A to B and B to A permeability of GRg3, CK, GRh2, PPD, GRg1, GRh1, and PPT in LLC-PK1-P-gp cells. (**C**) Kinetic parameters of CK, GRh2, PPD, and PPT in LLC-PK1-P-gp cells. (**D**) Concentration dependency for the B to A transport rate of CK, GRh2, PPD, and PPT was measured in LLC-PK1-P-gp cells. A line was generated from the Michaelis–Menten equation. Data are expressed as mean± standard deviation (*n* = 3).

Next, we measured the permeability (P_app_) of ginsenosides in CaCo-2 and LLC-PK1-P-gp cells, which have been widely used to investigate the absorption mechanism of drugs and new molecular entities [12,23], to determine the absorption mechanism relative to the different glycosylation sites (Figure 5A).

The apical to basal permeability (A to B P_app_) of highly glycosylated ginsenosides (i.e., GRb1, GRb2, GRc, GRd, GRg3, and GF2 of the PPD-type and GRe, GRf, GRg1, GF1, and GRh1 of the PPT-type) gradually increased as the sugar number decreased; however, they were below 0.3 × 10^−6^ cm/s. The B to A P_app_ also exhibited similar permeability to the A to B P_app_, and the efflux ratio (ER) of the ginsenosides was similar to unity in CaCo-2 cells (Figure 5A). These results suggested that glycosylated ginsenosides have limited permeability and that passive diffusion mechanisms are involved in the absorption process. However, among the 15 ginsenosides, CK, GRh2, PPD, and PPT showed significantly higher permeability. They were subject to an efflux system during the absorption process, as evidenced by an ER greater than 2 (Figure 5A,B). Moreover, the permeability of CK, GRh2, PPD, and PPT did not correlate with their sugar content.

Since the involvement of P-gp in the permeability of CK has been reported [21], we examined the role of P-gp in the transport of CK, GRh2, PPD, and PPT using a P-gp overexpression system (i.e., LLC-PK1-P-gp cells) [22]. The permeability of GRg3, GRg1, and GRh1 was very low, and the ERs for these ginsenosides were approximately 1.0 in LLC-PK1-P-gp cells, consistent with the results obtained in Ca*C*o-2 cells. The ER of GRh2 and CK in LLC-PK1-P-gp cells was even higher than that in Ca*C*o-2 cells, which may be attributed to the higher P-gp expression in LLC-PK1-P-gp cells compared with that in Ca*C*o-2 cells. The ER of PPD and PPT in LLC-PK1-P-gp cells was similar to that in Ca*C*o-2 cells (Figure 5B). This may be the result of the differential affinity of GRh2, CK, PPD, and PPT; thus, we determined the concentration dependency of the B to A transport rate of these ginsenosides.

The B to A transport rates of CK, GRh2, PPD, and PPT were concentration-dependent and saturated with increasing ginsenoside concentrations (Figure 5C,D). The intrinsic clearance (CL_int_) indicated that the transport activity of ginsenosides for P-gp was the highest in CK and decreased in the order GRh2 > PPT > PPD (Figure 5C,D), which was attributed primarily to the difference in the translocation capacity (V_max_) rather than the difference in the affinity for P-gp (K_m_). Considered together, the involvement of P-gp in CK, GRh2, PPD, and PPT may reduce intestinal absorption and may consequently decrease the oral BA of these ginsenosides.

## 4. Discussion

The beneficial role of LAB as a health supplement has become increasingly apparent, and LAB consumption has grown rapidly. Therefore, the co-administration of RGE and LAB is done frequently; the combined formulation of RGE and LAB has been introduced in the Korean health supplement market. In addition, ginsenoside metabolism is mediated by the gut microbiota, in which the involvement of LAB has been reported; These bacteria include *Lactobacillus* sp. [35,37,38,39,40], *Bifidobacterium* sp. [41,42,43,44,45,46], *Eubacterium* sp., *Fusobacterium* sp., *Bacteroides* sp., and *Microbacterium* sp. [43,44,45,46,47]. Therefore, the interaction between LAB and RGE and the resultant pharmacokinetics of ginsenosides warrant further investigation.

We previously reported that LAB supplementation (Lacto-fit^®^, Chong Kun Dang Health Care) increases the plasma concentrations of CK, PPD, and PPT in mice [12]. LAB supplementation for two weeks resulted in much higher AUC values for PPD (2.67-fold increase) and PPT (1.63-fold increase) compared to the control groups. This may be explained by the more efficient metabolic conversion to PPD from CK or GRh2 and to PPT from GRh1 compared to the gut microbiota-mediated intrinsic activity [12]. The incubation of ginsenosides with the LAB supplementation resulted in the highest metabolic activity for converting GF2 to CK and GRh2 to PPD, suggesting preferable deglycosylation at the C3 site of damarane structure mediated by the LAB supplementation. Considering the content of this LAB formulation (e.g., *Lactobacillus rhamnosus* and *Lactobacillus plantarum* 59%), deglycosylation at the C3 site may be mediated primarily by the *Lactobacillus* sp. [35,48,49]. Compared with these results, we found that the incubation of ginsenosides with the LAB formulation used in this paper resulted in the highest metabolic activity for converting CK to PPD, from GRg1 to GRh1, and from GF1 to PPT, suggesting that deglycosylation at the C20 site of the ginsenoside structure is mediated by LAB (Figure 4). Considering the major content of this LAB product (e.g., *Bifidobacterium bifidum* 50% and *Bifidobacterium longum* 30%), *Bifidobacterium* sp. is primarily involved in the deglycosylation at the C20 site of PPD-type and PPT-type ginsenosides, consistent with previous reports [49,50]. We also demonstrated the effects of facilitated fermentation of ginsenosides by *Bifidobacterium* sp. on the metabolism and pharmacokinetics of ginsenosides in humans. The plasma AUC of GRh2, CK, PPD, and PPT were 2.7-, 2.1-, 1.6-, and 3.5-fold, respectively, after LAB supplementation for 2 weeks (Figure 2 and Table 1). Compared to the in vivo human study, the in vitro formation rate of CK was weaker than that of GRh2, PPD, and PPT (Figure 1 and Figure 2). Therefore, the observed 2.1-fold increase in AUC value for CK may be attributed to the human intrinsic deglycosylation activity mediated by intestinal microbiota [13]. Consequently, LAB supplementation may activate the sequential deglycosylation of ginsenosides and activate the intrinsic deglycosylation activity. Taken together, these results suggest that LAB supplementation increases the plasma concentration of deglycosylated ginsenosides and may potentiate the therapeutic efficacy of these ginsenosides.

However, deglycosylated ginsenosides such as CK, GRh2, PPD, and PPT showed substrate specificity for P-gp, and the intrinsic clearance of these ginsenosides was 690, 502, 5.2, and 231 μL/min, respectively, in P-gp-overexpressing cells (Figure 5). These results suggested that P-gp-mediated efflux of these ginsenosides may limit the oral absorption of these deglycosylated ginsenosides. As previously reported [17], the relative BA of CK in P-gp null mice was 23.5-fold greater than that in wild-type mice, and oral BA was very low (4.3%) despite their higher lipophilicity and low molecular weight. Similarly, the involvement of P-gp and higher intrinsic clearance of GRh2 and PPT also contribute to their low oral BA (5% and 3.7%, respectively) [18,19]. PPD also showed P-gp-mediated efflux, but its intrinsic clearance was the lowest among the four ginsenosides tested. The results suggested the weaker involvement of P-gp compared with CK, GRh2, and PPT, which may contribute, in part, to the relatively higher oral BA of PPD (36.8–48.1% [15,19]). In addition, the K_m_ values for P-gp-mediated ginsenoside permeability were close to or even higher than 100 μM (Figure 5). This concluded that the efflux function could not be saturated by the oral dose of RGE. Taken together, these results suggest that LAB supplementation may increase the plasma exposure of deglycosylated ginsenosides, but the fold increase in AUC of CK, GRh2, PPD, and PPT in vivo human PK appeared to be lower than the increase in the in vitro metabolic activity through the involvement of P-gp in these ginsenosides.

We conducted a proof-of-concept study on the effect of LAB supplementation on the pharmacokinetics of ginsenosides in five human subjects. However, we should note the limitation of the small number of subjects enrolled in this study and the need to confirm our hypothesis in a large number of subjects. During the posthoc power analysis using our pharmacokinetic data for deglycosylated ginsenosides such as GRh2, CK, PPD, and PPT from RGE and RGE + LAB treatment groups, the statistical power of the existing AUC results was calculated as 91.1%, 88.6%, 79.4%, 65.9% for GRh2, CK, PPD, and PPT, respectively, with a significance level of 0.05 [51]. The minimum sample size for adequate study power (significance level of 0.05, statistical power of 80%) of our ginsenoside pharmacokinetic data from RGE and RGE + LAB treatment groups were estimated to be 2 to 6 subjects for GRh2, CK, PPD, and PPT [52]. The estimation indicated that our study design provided the minimum number of subjects to differentiate the significance between RGE and RGE + LAB groups for GRh2, CK, and PPD; however, it is necessary to expand the number of subjects for future confirmation studies to obtain statistical power for other ginsenosides detected in the plasma.

## 5. Conclusions

We performed a proof-of-concept study to demonstrate the effect of LAB supplementation on the formation rate of GRh2, CK, PPD, and PPT, deglycosylated ginsenoside metabolites, and the enhanced plasma exposure of these deglycosylated ginsenosides in human subjects. In vitro fermentation of RGE with LAB, mainly composed of *Bifidobacterium* sp., resulted in the highest formation rate of PPD and PPT, which do not exist in RGE. Consequently, LAB supplementation (1 billion CFU/day) for two weeks with RGE (39.6 mg of marker ginsenosides GRb1 + GRg3 + GRg1 and 104.1 mg of total ginsenosides/day) increases the plasma concentrations of deglycosylated ginsenosides including GRh2, CK, PPD, and PPT through the enhanced deglycosylation metabolic activity.

## Figures and Tables

**Table 1:** Pharmacokinetic parameters of ginsenosides in humans after repeated oral administration of RGE with or without repeated LAB treatment.

Ginsenosides	RGE			
	AUC (ng/mL·h)	C_max_ (ng/mL)	T_max_ (h)	MRT (h)
GRb1	184.6 ± 80.6	7.1 ± 3.2	5.3 ± 4.6	18.3 ± 1.1
GRb2	102.0 ± 51.0	3.9 ± 1.8	6.2 ± 4.0	18.5 ± 1.4
GRc	118.4 ± 58.2	5.1 ± 2.4	6.2 ± 4.0	18.0 ± 1.2
GRd	37.9 ± 12.6	2.1 ± 0.9	7.5 ± 5.8	18.2 ± 1.4
GRg3	94.1 ± 50.0	7.2 ± 4.8	7.2 ± 3.0	7.6 ± 2.8
GRh2	21.3 ± 10.1	2.1 ± 0.8	10.7 ± 0.6	17.0 ± 9.3
CK	57.9 ± 16.6	5.1 ± 2.2	8.0 ± 4.0	17.2 ± 4.5
PPD	129.5 ± 39.9	7.6 ± 1.7	16.0 ± 18.5	18.6 ± 5.0
PPT	59.9 ± 49.2	9.5 ± 4.8	17.4 ± 17.4	19.4 ± 10.5
Ginsenosides	RGE + LAB			
	AUC (ng/mL·h)	C_max_ (ng/mL)	T_max_ (h)	MRT (h)
GRb1	164.4 ± 77.3	6.2 ± 2.9	6.8 ± 1.1	16.9 ± 0.5
GRb2	88.6 ± 42.0	3.0 ± 1.4	7.2 ± 1.1	17.4 ± 0.1
GRc	109.6 ± 50.3	4.6 ± 2.3	8.2 ± 1.8	16.8 ± 0.3
GRd	24.1 ± 11.3 *	0.9 ± 0.4 *	7.6 ± 0.9	16.4 ± 0.8
GRg3	33.3 ± 21.7 *	4.8 ± 3.1	6.4 ± 3.3	6.7 ± 0.7
GRh2	57.1 ± 22.0 *	13.9 ± 12.8	10.6 ± 1.8	10.2 ± 1.6
CK	123.3 ± 43.1 *	12.6 ± 3.7 *	9.6 ± 1.5	13.1 ± 2.6
PPD	205.4 ± 81.7 *	12.5 ± 4.1 *	11.2 ± 1.6	16.6 ± 2.7
PPT	211.2 ± 134.0 *	18.0 ± 7.3 *	10.6 ± 1.8	13.1 ± 4.5

AUC, area under the plasma concentration-time curve from 0 to 24 h; C_max_, maximum plasma concentration; T_max_, time to reach C_max_; MRT, mean residence time. Data are expressed as mean ± standard deviation (*n* = 5). * *p* < 0.05 compared with the LAB group.

## Data Availability

Not applicable.
